# Prognostic significance of molecular characteristics of cerebrospinal fluid for non‐small cell lung cancer patients with leptomeningeal metastasis

**DOI:** 10.1111/1759-7714.13123

**Published:** 2019-07-10

**Authors:** Ning Li, Yutao Liu, Jianchun Duan, Boyan Yang, Hua Bai, Rui Sun, Lei Yu, Jie Wang

**Affiliations:** ^1^ Department of Comprehensive Oncology, State Key Laboratory of Molecular Oncolgy, National Cancer Center/National Clinical Research Center for Cancer/Cancer Hospital Chinese Academy of Medical Sciences & Peking Union Medical College Beijing China; ^2^ State Key Laboratory of Molecular Oncology, Department of Medical Oncology, National Cancer Center/National Clinical Research Center for Cancer/Cancer Hospital Chinese Academy of Medical Sciences & Peking Union Medical College Beijing China; ^3^ Department of Comprehensive Oncology, National Cancer Center/National Clinical Research Center for Cancer/Cancer Hospital Chinese Academy of Medical Sciences & Peking Union Medical College Beijing China

**Keywords:** *EGFR* mutation, leptomeningeal metastasis, liquid biopsy, non‐small cell lung cancer, survival

## Abstract

**Background:**

Studies in *EGFR*+ non‐small cell lung cancer (NSCLC) patients with leptomeningeal metastasis (LM) comparing survival rates and gene mutation detection with matched cerebrospinal fluid (CSF) and plasma are relatively scarce. We evaluated gene mutations, treatment strategies, and clinical outcomes in *EGFR*+ NSCLC patients with LM.

**Methods:**

We retrospectively reviewed gene mutation status in the CSF and plasma of 32 *EGFR*+ NSCLC patients with LM for prognostic significance.

**Results:**

The rate of LM disease control was significantly higher in patients that switched EGFR‐tyrosine kinase inhibitor (TKI) treatments, initiated EGFR‐TKIs, or received high‐dose EGFR‐TKI treatment than those who continued their current EGFR‐TKI treatment, received chemotherapy, or were not administered antitumor treatment (24/24, 100.0% vs. 1/8, 12.5%; *P* = 0.000). Overall survival was 27.0 months (95% confidence interval [CI] 19.0–37.5), median survival after LM was 7.0 months (95% CI 5.0–11.0), and median survival before LM was 17.0 months (95% CI 12–25.5). Median survival after LM was significantly shorter in patients with “worse” status (*n* = 7) than in those with “improved/stable” status (*n* = 25; 4.2 [95% CI 2.2–6.1] vs. 33.7 [95% CI 25.5–41.8] months, HR 10.114, 95% CI 0.29–1.37; *P* = 0.008).

**Conclusions:**

EGFR‐TKIs should be the priority course of treatment in *EGFR*+ NSCLC patients after a diagnosis of LM. Liquid biopsy in both plasma and CSF, as well as dynamic detection, play important roles in the direction of treatment for such patients.

## Introduction

Several kinds of solid tumors, including malignant melanoma and lung, breast, and gastric cancer are accompanied by leptomeningeal metastasis (LM) at some stage during disease progression. Among these cancers, non‐small cell lung cancer (NSCLC) accounts for approximately 40–50% of cases that include LM.^1^, ^2^ In recent years, the incidence of LM in patients with NSCLC has increased, especially in *EGFR*+ NSCLC patients, and 9.0% of EGFR+ NSCLC patients were diagnosed with LM.^3^, ^4^, ^5^ Whole brain radiotherapy (WBRT), systemic chemotherapy, and intrathecal chemotherapy (ITCT) are the traditional treatment choices for NSCLC patients with LM. However, there is no consensus on the optimal treatment strategy, and the efficacies of these treatments for LM patients remain unsatisfactory. Presently, LM is still closely associated with a poor prognosis and rapid deterioration in clinical situations. Therefore, the goal of LM treatment is to improve or stabilize neurological symptoms, which can improve quality of life and lead to improved survival rates.

An increasing number of reports have shown that NSCLC patients with *EGFR* gene mutations (*EGFR*+) tend to have more central nervous system (CNS) metastases, including both brain and leptomeningeal metastases after using of EGFR‐tyrosine kinase inhibitors (TKIs) in association with theuse of EGFR‐tyrosine kinase inhibitors (TKIs). There are several potential reasons for this phenomenon. Firstly, the survival of *EGFR*+ NSCLC patients has been significantly prolonged.^6^ Secondly, first (i.e. gefitinib) and second‐generation (afatinib) EGFR‐TKIs have poor penetration across the blood–brain barrier (BBB). The percentage of penetration ranges from 0.69% to 1.3%, which may provide a good opportunity for tumor cell growth in the CNS.^7^ Thirdly, as the treatment duration increases, the likelihood of the development of several secondary resistance mechanisms to EGFR‐TKIs also increases.^8^, ^9^, ^10^ Based on these considerations, the treatment options for *EGFR*+ NSCLC patients with LM generally include an intial high‐dose of first or second‐generation EGFR‐TKIs, or a switch to a different EGFR‐TKI.

*EGFR* mutations can be detected by analyzing circulating tumor DNA (ctDNA) in both plasma and cerebrospinal fluid (CSF), which provides an alternative to tumor biopsies for further study. With the widespread use of third generation EGFR‐TKIs, data on LM in NSCLC patients with *EGFR*+ is relatively scarce. We retrospectively assessed the diagnosis, treatment modes, and survival status of *EGFR*+ NSCLC patients with LM that had been enrolled in our hospital. We determined their gene mutation status by analyzing ctDNA extracted from peripheral blood, CSF, and tumor tissue samples.

## Methods

### Patient population

In this retrospective study, *EGFR*+ NSCLC patients diagnosed with advanced‐stage (stage IV) NSCLC between January 2016 and November 2018 were reviewed for diagnosis of LM. All LM patients needed to be diagnosed by magnetic resonance imaging (MRI) and/or by the detection of malignant cells in the CSF by cytopathological diagnosis. In the event that only atypical and/or suspicious cells were found in the CSF, patients were not diagnosed with LM.

All patients signed informed consent to participate in this study and gave permission to use their peripheral blood and CSF. The ethical committee of the Cancer Hospital, Chinese Academy of Medical Sciences and Peking Union Medical College approved the protocol.

### *EGFR* mutation detection

We ascertained the *EGFR* mutation status of patients in the following regions: *EGFR* driver mutations (exon 18 alteration, exon 19 deletion, exon 21 L858Arg, HER2 alterations), *EGFR* resistance mutations (exon 20 Thr790Met, exon 20‐C797s), or mutations in tumor suppressor genes of plasma and CSF ctDNA. *EGFR* mutation status was determined for all patients by analyzing ctDNA exracted from tumor samples and/or CSF. Paired CSF and plasma samples were collected at the same time from the majority of patients. Approximately 10 mL of CSF was collected by lumbar puncture for cytology examination and gene detection by amplified refractory mutation system (ARMS) or next‐generation sequencing (NGS). Additionally, 8 mL of plasma was collected for super AMRS or NGS analysis concurrently with the collection of CSF.

### Statistical analysis

Follow‐up continued until January 2019. The duration of investigation was calculated from the initial diagnosis of LM until death or the last date of follow‐up, with a minimum follow‐up period for statistical analysis set at one month. The primary outcome measurement was disease control of LM, which was evaluated using the following criteria: improved/stable, defined as clinical symptom improvement or maintaining a consistent state, and/or enhanced computed tomography (CT) or MRI examination showing decreased or stable lesion state; worse, defined as a worsening of clinical symptoms or observation of increased lesions on enhanced CT or MRI examination according to European Association of Neuro‐Oncology–European Society for Medical Oncology (EANO–ESMO) Clinical Practice Guidelines.^11^ Extracranial lesions that appeared to be LM were evaluated according to Response Evaluation Criteria in Solid Tumors (RECIST) version 1.1, and were defined as confirmed complete response (CR), partial response (PR), stable disease (SD), or progressive disease (PD) four weeks after LM diagnosis. Secondary outcomes were overall survival (OS), and survival duration before and after LM diagnosis. OS was defined as the duration from the diagnosis of lung cancer until the date of death or last follow‐up. Survival duration before LM was calculated from the diagnosis of lung cancer until LM diagnosis. Survival duration after LM was calculated from the date of LM diagnosis to the date of death.

Comparisons of categorical and continuous variables were performed using the χ^2^ test and independent *t*‐test, respectively. Survival analyses were performed according to the Kaplan–Meier method and tested for significance with the log‐rank test. Comparisons between subgroups were made using a Cox proportional hazards model and Wald 95% confidence intervals (CIs), where appropriate. Statistical analyses were performed using SPSS version 19.

## Results

### Characteristics of leptomeningeal metastasis (LM) in non‐small cell lung cancer (NSCLC) patients

The medical records of 32 patients with a diagnosis of *EGFR*+ advanced‐stage NSCLC with LM were reviewed. LM was diagnosed by both MRI and CSF cytology in 20 patients (62.5%), only by CSF cytology in 10 patients (31.3%), and only by MRI in two patients (6.3%) (Table 1).

**Table 1:** Clinical characteristics of patients with LM from NSCLC

Characteristics	*n* = 32	*P*
Gender (male/female)		
Male	17	
Female	15	
Median age, year (range)		
< 60	22	
≥ 60	10	
Histopathology		
Adenocarcinoma	30	
Unspecified non‐small cell carcinoma	2	
Median time from cancer diagnosis to LM, months		0.046
< 12	12	
≥ 12	20	
Yes	28	0.000
No	4	
ECOG PS		
0–2	28	0.000
3–4	4	
Extracranial SD/PR		
Yes	25	0.000
No	7	

ECOG PS, Eastern Cooperative Oncology Group performance status; LM, leptomeningeal metastasis; NSCLC, non‐small cell lung cancer; PR, partial response; SD, stable disease.

The median time from NSCLC diagnosis to LM diagnosis with *EGFR*+ was 17.0 months (95% CI 12.0–25.5, range: 0–88), and there were significant differences in duration between patients diagnosed within 12 and over 12 months. (20/32, 62.5%; *P* = 0.046). A total of 28 patients had brain metastasis (BM) before LM or at the same time as LM diagnosis (87.5%, *P* = 0.000). Eastern Cooperative Oncology Group performance status (ECOG PS) was evaluated at the time of LM diagnosis. The ECOG PS of 28 patients was 0–2 (87.5%; *P* = 0.000), and 25 patients were assessed with SD/PR of extracranial tumors (78.1%; *P* = 0.000) (Table 1).

### *EGFR* mutation status in histopathology, cerebrospinal fluid, and plasma DNA

**Figure 1:**
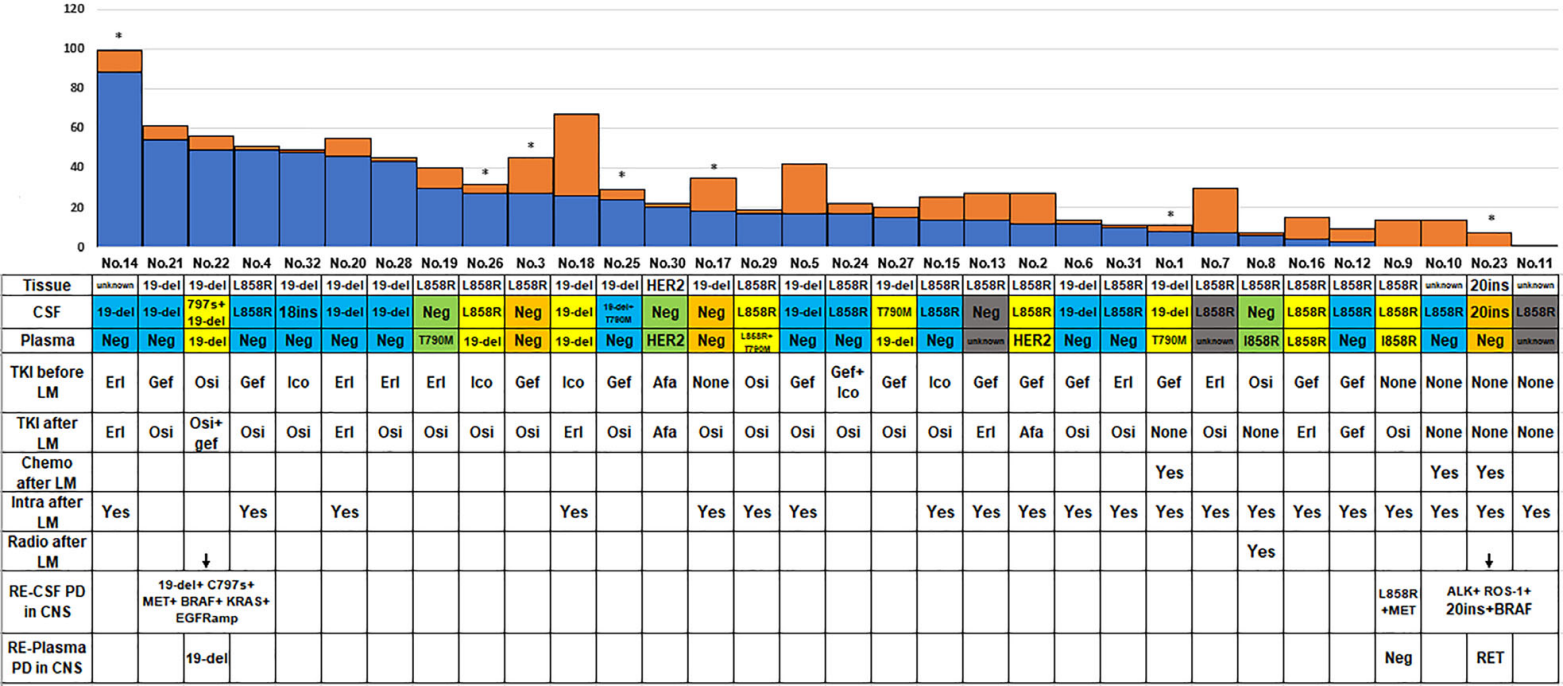
Treatment status and *EGFR* mutations in histopathology, cerebrospinal fluid (CSF), and plasma DNA. *Patient died. Afa, afatinib; Eri, erlotinib; Gef, gefitinib; Ico, icotinib; m, months; Osi, osimertinib. (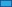) Patients with *EGFR* driver mutations and/or T790M negative in plasma but positive in CSF. (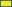) Patients with *EGFR* driver mutations and/or T790M positive in both plasma and CSF. (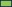) Patients with *EGFR* driver mutations and/or T790M positive in plasma but negative in CSF. (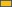) Patients with *EGFR* driver mutations and T790M negative in both plasma and CSF. (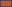) Patients with *EGFR* driver mutations and T790M unknown in plasma. (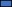) From cancer diagnosis to LM diagnosis (m) and (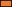) After LM diagnosis (m).

Twenty‐nine patients (90.6%) underwent *EGFR* mutation analysis and histopathology before initial treatment: 12 (41.4%) had exon 19 deletions, 15 (51.7%) had exon 21 L858R, 1 (3.4%) had an exon 20 insertion, and 1 patient (3.4%) had an *ERBB2* alteration (Fig 1).

All 32 patients underwent identical *EGFR* driver mutation detection analysis on ctDNA extracted from the CSF after LM diagnosis: 20 (62.5%) were tested by ARMS, and 12 were tested by NGS. The results revealed: 13 patients (37.5%) had exon 21 L858R mutations; 8 (25.0%) had exon 19 deletions; 6 (18.8%) had wild‐type *EGFR*; 1 (3.1%) had a T790M mutation; 1 (3.1%) had an exon 19 deletion and T790M mutation; 1 (3.1%) had an exon 20‐C797S mutation and exon 19 deletion; 1 (3.1%) had an exon 20‐770 alteration; and 1 patient (3.1%) had an exon 18 alteration (Fig 1).

Among the 12 patients whose mutations were detected by NGS in CSF, 2 (16.7%) had T790M mutations, 6 (50.0%) had TP53 alterations, and 7 (58.3%) had *EGFR* driver mutations (Table 2).

**Table 2:** Gene mutation status detected by NGS in CSF

Gene mutation	*n*	Percentage
*EGFR* exon 19 + *TP53*	1	8.30%
*EGFR* exon 20–797s + exon 19	1	8.30%
*EGFR* exon 20–770 + *TP53*	1	8.30%
*EGFR* exon 21 L858R + *TP53*	2	16.70%
*EGFR* exon 19 + T790M + *TP53*	1	8.30%
T790M	1	8.30%
*EGFR* exon 19	1	8.30%
*EGFR* exon 21	2	16.70%
*TP53*	1	8.30%
*EGFR* exon 18	1	8.30%

CSF, cerebrospinal fluid; NSG, next generation sequencing.

Analysis of *EGFR* mutations via ARMS versus NGS in CSF showed different results. The detection rate of *EGFR* T790M and exon 20 mutations was significantly higher by NGS than by ARMS (2/12, 16.7% vs. 0/20, 0.0%, *P* = 0.133; 2/12, 16.7% vs. 0/20, 0.0%, *P* = 0.133, respectively).

Among the 32 patients, 29 underwent mutation analysis after LM diagnosis in plasma paired with CSF. Their results were as follows: 16 patients (16/29, 55.2%) had wild‐type *EGFR*; 4 (4/29, 13.8%) had *EGFR* exon 19 deletions; 3 (3/29, 10.3%) had *EGFR* exon 21 L858R mutations; 2 (2/29, 6.9%) had *HER2* alterations; 2 (2/29, 6.9%) had *EGFR* T790M mutations; 1 (1/29, 3.4%) had *ROS‐1* alterations; and 1 patient (1/29, 3.4%) had *EGFR* exon 21 L858R and T790M mutations (Fig 1).

**Figure 2:**
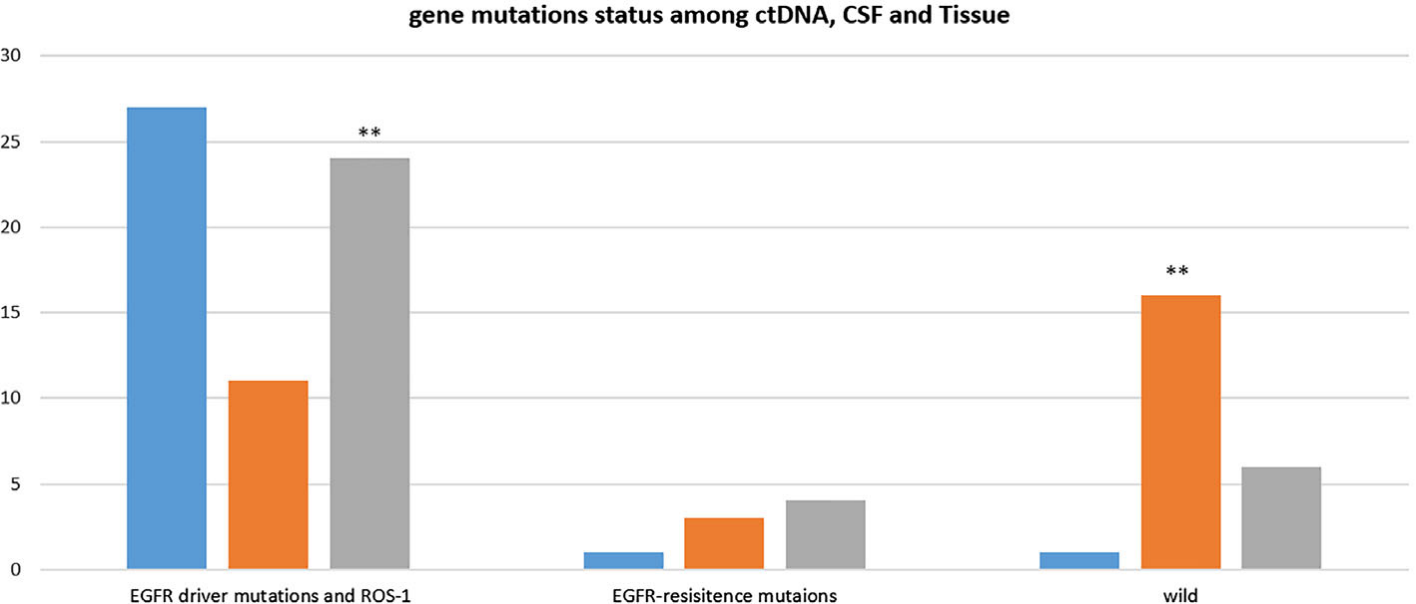
Positive detection among plasma, cerebrospinal fluid (CSF), and tissue.*P = 0.003 *EGFR* driver mutations and *ROS‐1* plasma versus CSF. **P = 0.005 wild plasma versus CSF. ctDNA, circulating tumor DNA. (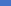) Tissue (*n* = 29), (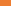) Plasma (*n* = 29), and (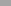) CSF (*n* = 32).

In summary, 11 patients showed *EGFR* driver mutations and *ROS‐1* in plasma, while 24 patients showed *EGFR* driver mutations in CSF (11/29 vs. 24/32; *P* = 0.003). No mutations were detected in the plasma and CSF of 16 and 6 patients, respectively (16/29 vs. 6/32; *P* = 0.005) (Fig 2).

### Treatment of *EGFR*‐mutated NSCLC patients with LM

At the time of LM diagnosis, 27 patients (84.4%) were treated using a specfic EGFR‐TKI. Five different regimens were used during follow‐up treatment for LM according to the personal and specific conditions of each patient: EGFR‐TKIs, chemotherapy, WBRT, ITCT, and TMZ. EGFR‐TKI treatment included: continuation of current EGFR‐TKI treatment, initiation of EGFR‐TKI treatment, switching from one EGFR‐TKI treatment to another, and high‐dose EGFR‐TKI treatment (Table 3). There was a significant difference in the rate of LM disease control between patients that switched EGFR‐TKI treatments, intiated EGFR‐TKI treatment, or received high‐dose EGFR‐TKI treatment and those that continued with their current EGFR‐TKI treatment, received chemotherapy, or were not administered antitumor treatment (24/24, 100.0% vs. 1/8, 12.5%; *P* = 0.000) (Table 3). Among this group, 21 patients (21/32, 65.6%) were treated with combined ITCT (Table 4), 1 patient (3.1%) with TMZ, and 1 patient (3.1%) with radiotherapy. Subgroup analysis of the rate of LM disease control indicated no significant difference between patients administered third‐generation EGFR‐TKIs with or without ITCT (8/9, 88.9% vs. 10/10, 100.0%, respectively; *P* = 0.474) (Table 5).

**Table 3:** Treatment in *EGFR*‐mutated NSCLC patients with LM

Treatment before LM		Treatment after LM	Disease control of LM after 1 month (*n*)	
(Targeted therapy or chemotherapy)	*n*	(Targeted therapy or chemotherapy)	Worse	Improved/Stable
Chemotherapy	1	Osimertinib	0	1
No treatment	4	Osimertinib	0	1
		Chemotherapy	1	1
		No treatment	1	0
Gefitinib or icotinib	17	Chemotherapy	1	0
		Gefitinib	1	0
		Erlotinib	0	3
		Afatinib	0	1
		Osimertinib	0	11
Erlotinib	6	Doubled erlotinib	0	1
		Osimertinib	0	5
Afatinib	1	Afatinib	1	0
Osimertinib	3	Osimertinib	1	0
		Osimertinib and gefitinib	0	1
		No treatment	1	0

LM, leptomeningeal metastasis; NSCLC, non‐small cell lung cancer.

**Table 4:** Response of LM treated by ITCT or not

Combined with ITCT	*n*	Improved/Stable	*P*
Yes	21	(16/21) 76.2%	0.078
No	11	(11/11) 100%	

ITCT, intrathecal chemotherapy; LM, leptomeningeal metastasis.

**Table 5:** Response of LM after EGFR‐TKI with or without ITCT

		Systemic treatment after LM						
Response		EGFR‐TKIs^1st^	EGFR‐TKI^2nd^	EGFR‐TKI^3rd^	Chemotherapy	No treatment	*N*	*P*
Improved/Stable	No ITCT	0	1	10	0	0	25	0.000
	ITCT	5	0	8	1	0		
Worse	No ITCT	0	0	0	0	0	7	
	ITCT	1	1	1	1	3		

ITCT, intrathecal chemotherapy; LM, leptomeningeal metastasis; TKI, tyrosine kinase inhibitor.

There was a significant difference in the rate of LM disease control between patients with *EGFR* driver and/or T790M‐positive mutations and those without *EGFR* driver mutations or T790M‐negative (9/10, 90.0% vs. 0/2, 0.0%, respectively; *P* = 0.007) (Table 6).

**Table 6:** Response of LM according to different gene mutations in CSF

Response	*n*	Improved/Stable	Worse	*P*
Gene mutations by NGS				
*EGFR* driver mutations and/or T790M				
Positive	10	9	1	0.007
Negative	2	0	2	
Tumor‐suppressor gene mutations				
Positive	6	5	1	0.505
Negative	6	4	2	
*EGFR* driver mutations by ARMS				
*EGFR* exon 19	6	5	1	0.966
*EGFR* exon 21	9	7	2	
Wild	5	4	1	

ARMS, amplified refractory mutation system; CSF, cerebrospinal fluid; LM, leptomeningeal metastasis; NSG, next‐generation sequencing.

### Survival and response of *EGFR*‐mutated NSCLC patients with LM

**Figure 3:**
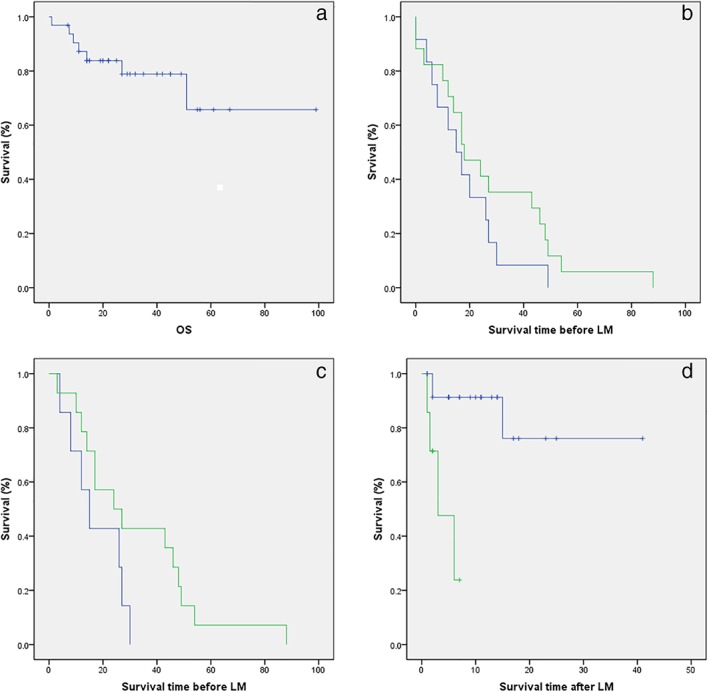
Kaplan–Meier curves of (**a**) overall survival (OS) in *EGFR*‐mutated non‐small cell lung cancer patients with leptomeningeal metastases (LM); (**b**) survival duration before LM according to *EGFR* driver mutations and/or T790M in plasma (hazard ratio [HR] 0.63, 95% confidence interval [CI] 0.29–1.37; *P* = 0.211), (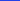) Plasma positive and (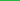) plasma negative; (**c**) survival duration before LM according to *EGFR* driver mutations and/or T790M in plasma using first‐generation EGFR‐tyrosine kinase inhibitors (HR 2.41, 95% CI 0.87–6.70; *P* = 0.078), (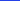) Plasma positive and (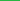) plasma negative; and (**d**) survival duration after LM diagnosis according to LM disease control status (HR 10.114, 95% CI 0.29–1.37; *P* = 0.008), (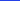) Improved/stable and (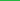) worse.

Seven (7/32, 21.9%) of the *EGFR*+ NSCLC patients with LM died before their follow‐up appointments and the one‐year survival rate was 59.4% (19/32). The OS of all 32 patients was 27.0 months (95% CI 19.0–37.5) (Fig 3).

The median survival duration before LM in all 32 patients was 17.0 months (95% CI 12–25.5): 15.0 months (95% CI 4.0–27.0) in 9 patients with *EGFR* driver and/or T790M positive mutations in both plasma and CSF; 20.5 months (95% CI 12.0–48.0) in 14 patients with *EGFR* driver mutations and/or T790M negative in plasma but positive in CSF; 20.0 months (95% CI 6.0–30.0) in 3 patients with *EGFR* driver and/or T790M positive mutations in plasma but negative in CSF; and 18.0 months (95% CI 0.0–27.0) in 3 patients with *EGFR* driver mutations and T790M negative in both plasma and CSF. The median survival duration in 17 patients with *EGFR* driver mutations and/or T790M negative in plasma was longer than in 12 patients with *EGFR* driver and/or T790M positive mutations in plasma (18.0 [95% CI 12.0–27.0] vs. 16.0 months [95% CI 7.0–26.5]; HR 0.63, 95% CI 0.29–1.37; *P* = 0.211) (Fig 3b, Table 7). Subgroup analysis of patients who acquired resistance to first‐generation EGFR‐TKIs revealed that median survival in 14 patients with *EGFR* driver mutations and/or T790M negative in plasma was longer compared to 7 patients with *EGFR* driver and/or T790M positive mutations in plasma (24.0 [95% CI 5.7–42.3] vs. 15.0 months [95% CI 7.3–22.7]; HR 2.41, 95% CI 0.87–6.70; *P* = 0.078) (Fig 3c).

**Table 7:** Systemic treatment before LM according to *EGFR* status in plasma

	Systemic treatment before LM						
EGFR and/or T790M	EGFR‐TKIs^1st^	EGFR‐TKI^2nd^	EGFR‐TKI^3rd^	Chemotherapy	No treatment	*n*	*P*
Positive	7	1	3	0	1	12	0.083
Negative	14	0	0	3	0	17	

LM, leptomeningeal metastasis; TKI, tyrosine kinase inhibitor.

The median survival duration after LM of all 32 patients was 7.0 months (95% CI 5.0–11.0): 11.4 months (95% CI 5.5–20.6) in 9 patients with *EGFR* driver and/or T790M positive mutations in both plasma and CSF; 7.2 months (95% CI 4.1–11.0) in 14 patients with *EGFR* driver mutations and/or T790M negative in plasma but positive in CSF; 4.5 months (95% CI 1.5–10.0) in 3 patients with *EGFR* driver and/or T790M positive mutations in plasma but negative in CSF; and 14.0 months (95% CI 7.0–18.0; *P* = 0.477) in 3 patients with *EGFR* driver mutations and T790M negative in both plasma and CSF.

Compared to patients with improved/stable LM (*n* = 25), the median survival duration after LM was significantly shorter in patients with worse LM (*n* = 7;33.7 [95% CI 25.5–41.8] vs. 4.2 months [95% CI 2.2–6.1]; HR 10.114, 95% CI 0.29–1.37; *P* = 0.008) (Fig 3d).

## Discussion

We monitored a group of *EGFR*+ NSCLC patients with LM and assessed the status of their varied *EGFR* mutations in paired plasma and CSF samples. Our results showed that *EGFR*+ NSCLC patients with LM had better response rates when they switched EGFR‐TKI treatments or received high‐dose EGFR‐TKI treatment after LM diagnosis. Our results also revealed that more mutated genes were found in CSF samples, and to some extent there were differences in the gene mutation status among tissues, plasma, and CSF. This result demonstrates the importance of detecting gene mutations in both CSF and plasma by NGS after LM diagnosis.

In previous studies, researchers reported that NSCLC patients not administered systemic chemotherapy, chemotherapy, or EGFR‐TKI treatment as a basic option after LM diagnosis only survived three months.^12^, ^13^ Kuiper *et al*. reported a survival duration after LM diagnosis of only 3.1 months in *EGFR*+ patients, as third‐generation EGFR‐TKIs, such as osimertinib, were not available for clinical treatment at the time.^4^ In our study, the median survival after a diagnosis of LM in the *EGFR*+ group was 7.0 months, which included patients who received high doses of first‐generation EGFR‐TKIs or switched EGFR‐TKI treatment (i.e. from first‐generation to second or third‐generation EGFR‐TKIs). Because *EGFR*+ patients had higher ECOG PS scores at the time of LM diagnosis, patients with unspecific symptoms, such as headaches and dizziness, need to have their LM status taken into consideration.

Because of the BBB, the concentrations of available first and second‐generation EGFR‐TKIs in CSF, such as gefitinib, erlotinib, and afatinib, are considerably lower compared to the concentration in extracranial lesions.^7^ When the extracranial concentration of EGFR‐TKIs is increased, a higher concentration in the CSF can be achieved, which can have a positive therapeutic effect.^14^ Under this treatment strategy, drug toxicity needs to be monitored and deemed acceptable. Reports have shown that this treatment strategy for *EGFR*+ NSCLC patients with LM has produced various results.^15^, ^16^ Kuiper *et al*. reported that 12 *EGFR*+ NSCLC patients with LM treated with high dose first‐generation EGFR‐TKIs after LM diagnosis achieved survival after LM of only 2.4 months.^4^

Afatinib is a second generation EGFR‐TKI. A recent study showed that if patients experienced disease progression after treatment of standard doses of erlotinib or gefitinib, 66% of them could achieve disease control when they switched to afatinib.^17^ In our study, one patient exhibited CNS disease control with afatinib after switching from gefitinib and achieved survival of 12 months. In addition, the third‐generation EGFR‐TKI osimertinib is reported to be active in the CNS.^18^, ^19^, ^20^ In this retrospective study, the LM disease control of all 19 patients was either improved or stable after switching from gefitinib or erlotinib to osimertinib, regardless of whether the T790M mutation was negative or positive. Patients that switched EGFR‐TKI treatments, intiated EGFR‐TKI treatment, or received high‐dose EGFR‐TKI treatment had better LM disease control status. Furthermore, in our study, improved and stable LM disease control status was predictive of better clinical outcomes.

In recent years, ITCT has been a traditional treatment choice for NSCLC patients with LM, but evidence of its efficacy is limited. In a sample of 105 NSCLC patients with LM, ITCT use resulted in OS of only 3.0 months. Furthermore, varying symptoms, such as headaches, altered mental state, and cauda equine, showed relatively low response rates, ranging from 42% to < 10%, and negative conversion of CSF cytology could not prolong OS. In our study, only 16 out of 21 patients administered ITCT achieved disease control (76.2%, *n* = 16), whereas all 11 patients not administered ITCT achieved disease control, which could be associated with the small sample size. Third generation EGFR‐TKIs combined with ITCT did not achieve a higher rate of LM disease control compared to third genenration EGFR‐TKIs alone. Unfortunately, there are no satisfactory survival results available related to ITCT.

Radiotherapy is another traditional treatment after a diagnosis of LM; however, there is a lack of evidence of the efficacy of radiotherapy in NSCLC patients with LM, and the toxicity associated with whole cerebrospinal radiotherapy can cause high mortality. In a report of 125 patients with LM, no difference in survival was observed between patients treated with or without WBRT.^13^ In our study, this treatment strategy could not be incorporated into the analyses because only one patient was administered radiotherapy after an LM diagnosis. Some reasons for the low use of radiotherapy in our study stem from the inconclusive efficacy of radiotherapy for LM, and the fact that some of the patients were administered radiotherapy for brain metastasis prior to LM diagnosis.

In our study, SD or PR was achieved in more extracranial tumors than in a previous study of unselected NSCLC patients with LM which reported that PD was associated with extracranial tumors. It is possible that extracranial tumors and LM arise from different mechanisms.^21^ Because of the BBB, CSF circulation is isolated from the blood circulation system, which may be one of the reasons why gene mutations in CSF are different to those in plasma. In our study, only 9.4% of patients had paired plasma and CSF samples (*n* = 3) with gene mutations in common. In 12 patients diagnosed by NGS, more types of gene mutations in CSF were associated with both secondary resistance genes and suppressors, in addition to other *EGFR* driver mutations. Therefore, detecting genomic alterations in CSF directly, especially by NGS, is a feasible method to represent the genetic status of intercranial lesions. Similarly, ctDNA in plasma provides an alternative to tumor samples for *EGFR* mutation analysis, and plasma gene mutations showed greater advantages than solid tumor cells for revealing the genetic landscape of primary and metastatic lesions, which are relatively limited. In the BENEFIT clinical trial, *EGFR* mutations reappeared in ctDNA with plasma samples when the disease progressed in 46% (56/123) of patients.^22^ In our study, 37.9% (11/29) of patients had *EGFR* driver mutations and *ROS‐1* in plasma when LM appeared, which is relatively low compared to the results of the BENEFIT analysis. However, in our study, more patients had *EGFR* driver mutations in CSF and more without *EGFR* driver mutations in plasma when LM was diagnosed, which is relatively high compared to the BENEFIT results. Therefore, LM might have a different mechanism from extracranial progression, and further studies need to be conducted to verify this possibility. Gene mutation status in an individual patient can be spatiotemporally heterogeneous, and in this case, it is necessary to detect multiple lesions simultaneously. The median survival duration before LM of 17 patients with *EGFR* driver mutations and/or T790M negative in plasma was 18.0 months, which was longer than in patients found to be T790M mutation positive in plasma. Subgroup analysis indicated that the median survival before LM of patients with *EGFR* driver mutations and/or T790M mutation negative in plasma and administered first‐generation EGFR‐TKIs was 24.0 months, which was longer than that when found positive in plasma. This result suggests that patients without gene mutations in plasma could have longer survival before LM, although the difference between subgroups was not significant. A similar result was observed in the BENEFIT study.^22^

Acquired resistance to EGFR‐TKIs is a common clinical problem, and the T790M mutation has been confirmed by several studies to be one of the most important biomarkers of acquired resistance.^10^ In our study, T790M was identified in two patients (2/12, 16.7%) in CSF by NGS, which is a lower rate than previously reported.^23^ However, none of the patients (0/20, 0%) had T790M in CSF when assessed by ARMS, which may be related to the technology used. The current standard treatment for T790M‐mediated resistance after confirmation of disease progression is third‐generation EGFR‐TKIs, such as osimertinib. In our study, the rate of disease control in patients (9/10, 90.0%) with *EGFR* driver mutations and/or T790M was significantly higher than in patients (0/2, 0%) without *EGFR* driver mutations and T790M. However, acquired resistance to osimertinib was also observed, and the molecular mechanisms of resistance (such as *EGFR*‐C797S, *BRAF*‐V600E, *MET*amp, and *ERBB2*amp) are not yet fully understood. In our study, one patient developed an *EGFR*‐C797S resistance mutation in CSF accompanied with an *EGFR* exon 19 deletion (1/12, 8.3%) after osimertinib treatment. Therefore, NGS with more advanced, targeted, ultra‐deep sequencing in CSF, which could more effectively monitor the gene mutation status and provide clinicians more opportunities to adjust EGFR‐TKI therapy for patients after acquired resistance to classic EGFR‐TKIs is needed.

*TP53* is a common tumor‐suppressor gene and is more highly expressed in patients whose disease transforms to SCLC (82%), which is another factor for EGFR‐TKI resistance in lung adenocarcinoma.^24^ In our study, *TP53* (6/12, 50%) was uniquely identified in CSF ctDNA, but there was no significant difference in disease control between patients with or without *TP53*. This result suggests that *TP53* may play a role in the progression of LM, but larger sample size studies are required to further explore this possibility.

In our study, three patients had re‐biopsies after presenting with clinical deterioration in the CNS. One patient was administered eight ITCT treatments and systemic treatment for five months, and after clinical deterioration in the CNS was observed, re‐biopsy detected alterations of *ALK*, *ROS‐1*, *EGFR* exon 20 insertions, and *BRAF* in the CSF. After taking alectinib for one month, the LM symptoms in the CNS improved. Another patient was found to have L858R in both CSF and plasma when diagnosed with LM, and achieved disease control for 14 months until clinical deterioration in CNS was observed. Re‐biopsy revealed *EGFR* L858R and *MET* alterations in the CSF. Another patient with *EGFR* exon 19 deletion in plasma and *EGFR* exon 19 deletion and exon 20 insertion‐C797S in CSF when diagnosed with LM achieved disease control for seven months until the appearance of clinical deterioration in CNS. Re‐biopsy detected coexisting *EGFR* exon 19 deletion, *EGFR* exon 20 insertion‐C797S, *MET*, *BRAF*, *KRAS*, and *EGFR*amp in the CSF. These results collectively suggest that it is important to re‐biopsy in clinical practice after disease progression in order to find any new mutations and alter therapeutic strategies.

Our study has several limitations. First, the study was retrospective with a small sample size, particularly regarding the use of NGS. Second, the rate of dynamic monitoring of ctDNA in CSF and plasma during treatment after LM was low. We plan to investigate the efficacy of EGFR‐TKIs in prospective trials based on *EGFR* mutations detected by other methods, such as NGS, and concentrate on dynamic detection.

In conclusion, we describe a cohort of *EGFR*+ NSCLC patients with LM. The median survival duration after diagnosis of LM was 7.0 months. EGFR‐TKIs should be considered priority treatment, as they are associated with superior survival in NSCLC patients after a diagnosis of LM. Tissue and liquid biopsies from multiple sites, as well as dynamic detection by NGS, play important roles in determining treatment strategies for *EGFR*+ NSCLC patients with LM.

## Disclosure

No authors report any conflict of interest.
